# Use of Opium in Children

**Published:** 1858-11

**Authors:** Thomas Pollard

**Affiliations:** Richmond, Va.


Vol. IV.] NOVEMBER, 1858. [No. III.

### ORIGINAL COMMUNICATIONS.

### ARTICLE I.

*Use of Opium in Children.* By Thomas Pollard, M. D., Richmond, Va.

There are two classes of medical practitioners with regard to the use of Opium in children. One uses it cautiously, aware of its dangers ; the other discards it altogether, magnifying we think, most unwarrantably, the objections to its exhibition in young subjects. Its dangers and advantages we propose to speak of, candidly and fairly. That it has dangers in its use with infants cannot be denied, but so has every remedy of any utility or power when applied to the treatment of infantile diseases. Mercury, Blisters, Tartar Emetic, are all dangerous when incautiously and carelessly prescribed in this class of patients, but why Opium has been so much reproached and condemned in children, we are at a loss to know, unless it is because it is a remedy of known power and activity, and has been improperly used. We do not discard the first named remedies from our Therapeutics, and there is no good reason why we should ostracize Opium, which, when cautiously exhibited, is equally as efficacious in the child as in the adult.

The system of the child is endowed with more susceptibility and irritability than that of the grown person, and on this account the composing effect of Opium is in some diseases more imperatively demanded in the former, than the latter. While at the same time this susceptibility requires that we should administer the remedy with more caution in cases of children. The brain of the child is supplied with more blood than that of the adult, and is of course more prone to congestion, and this is another reason why the remedy should be more cautiously exhibited to children, but these congestions are often dependent upon irritability and excitement of the cerebral organ, and are prevented by the timely exhibition of Opium, which by allaying this excitement, puts a stop to the first symptom in the chain of morbid action leading on to congestion and inflammation.

The child requires more sleep than the grown person, and the system is sooner worn down for the want of it. If the child does not sleep, its irritability becomes extreme, which in its turn is followed by convulsions and coma, or emaciation and exhaustion, by real or simulated congestion, or by actual or apparent Hydrocephalus. Where the child does not sleep, and suffers from constant pain and restlessness, it is surely then the part of wisdom and sound practice to procure rest by Opium cautiously administered. Opium, may and probably has, sometimes produced convulsions in children, but we believe it has, by allaying the irritability, and nervous excitement, more frequently prevented them. It has done harm in cases of children as have other active remedies, but we believe the good accomplished by Opium in this class of patients has been a great deal more than the evil resulting, even including the incautious administration of the drug. One great cause of the bad effects attributed to Opium in children has been the exhibition of too large doses of this drug. Dr. Dewees gives the following rules for the exhibition of Laudanum to children, viz:

"Give ½ drop for a child under 10 days old, 1 drop for a child over 10 days old and up to 1 month, 1½ to 2 drops, from that period to 3 months, and 3 drops from this to 9 months, these doses, he adds, are prescribed for children altogether unused to the drug, and the power of bearing more may be rapidly increased." These rules have no doubt been much followed by the profession, as "Dewees on Children" has been a standard work, and doubtless has done much harm. No wonder we have heard of deaths among children from the use of Opium when it has been the custom to give one drop of Laudanum to a child within the month and 3 drops from the 3d to the 9th month. Dr. Clarke says $\frac{1}{2}$ drop of Syrup of Poppies has proved fatal to *very young children* in a few hours. $\frac{1}{2}$ drop Syrup of Poppies is equal to $\frac{1}{16}$ grain of Opium, or $1\frac{1}{4}$ drops of Laudanum at the least. Wm. Marley says he knew an infant nearly poisoned by $\frac{1}{2}$ teaspoonful of Paregoric, which is nearly equal to $\frac{1}{8}$ gr. of Opium, or about $2\frac{1}{2}$ drops of Laudanum. Dr. Bard says he once knew an infant of several months old, killed by 10 drops of Laudanum, (what he might expect,) and one brought into great danger by 2 drops. Prof Hamilton relates two cases in which 4 drops proved fatal to children some months old*. I have recently been called to a child of 3 weeks old narcotized by $\frac{1}{2}$ drachm Paregoric. Now all these are large doses, unjustifiably large doses to begin with. Modern practitioners use opiates more cautiously, commencing with smaller doses, while some, as before mentioned, frightened by these accidents, discard them altogether. In commencing the use of opiates in very young subjects we should begin with very small doses, and increase the dose very gradually, if necessary to be increased, until the desired effect is produced. We must feel our way cautiously, for we will meet with peculiarities of constitution, and peculiarities of states of the system which bear opiates badly, and we must test these peculiarities very carefully. It is difficult to lay down any rule as to the exact size of the dose suited to the different ages. The writer has lately seen a case where 3 drops of Paregoric given twice, at an interval of two hours, composed an infant of 10 days old, producing quiet and a refreshing nights rest and doing the greatest good ; the child

*Vide, Beck on "Infantile Therapeutics." Page 16.

had "Cholera Infantum," had not slept the night before or during the day; the surface was cold, and the patient seemed almost moribund. The morning following, the greatest improvement had taken place; in this case one drop was first given, and the desired effect not being produced, the quantity was increased to 3 drops. I may here mention a case in which the greatest good was produced by the use of Opium in "Laryngismus Stridulus" in a child 6 months old: Every remedy of the Materia Medica had been used, the child grew worse, and finally, general convulsions supervened. The patient was carried home from the city, by boat to one of the lower counties of the State. The day it left the city it had general convulsions to the number of 10; after getting home, the father, an intelligent physician, determined to give the child Laudanum, more to relieve it of its sufferings, than with any hope of permanent good; the dose was 3 drops of Tinct. Opii. repeated "pro re nata," all other treatment being abandoned. The effect was most happy, and the father, two or three weeks after the commencement of the opiate treatment, informed me that the child was improving rapidly with every prospect of entire recovery.

The rules laid down in the "Physician's Visiting List" to regulate the doses of medicine for children is, at least, a safe one, as applied to the exhibition of opiates; this rule it will be recollected is "diminish the dose in proportion to the age, to the age increased by 12," thus, at 2 years of age it would be $\frac{2}{2+12}$ or $\frac{1}{7}$ of what it would be for an adult, and applying this to Opium, and putting the average dose for an adult at 1 grain we would have $\frac{1}{7}$ grain, or about 3 drops of Laudanum for this age, at *one month* it would be $\frac{1}{12}$ divided by $\frac{1}{12+12}$ or $\frac{1}{12}$ divided by $12\frac{1}{12}$ which would be about $\frac{1}{144}$ parts which would be $\frac{1}{144}$ part of a gr. of Opium, or $\frac{25}{144}$, or about $\frac{1}{6}$ of a drop of Laudanum, putting 25 drops as equivalent to a grain of Opium. This probably would be about the proper dose to commence with in a child of one month old and the rule will be found to be a safe one, and a useful one in regulating the doses of this agent for infants. It is not safe, however, to begin with *average* doses for newly born infants. To practice safely we must begin with the smallest doses and gradually feel our way. In the very young child, we are entirely ignorant of the manner in which so active an agent as Opium will affect the subject. If we begin with these small doses, we have a guide to make the necessary increase in the quantity to be given.

Prof. Merce, of the Childrens Hospital, at Pesth in Germany, although a decided advocate for the use of Opium in children, gives it with much caution. He says, "to the newly born babe, until it is a week old, I have rarely prescribed it. From the 2d to the 3d week the medium dose is $\frac{1}{120}$ grain of Opium, (or about $\frac{1}{6}$ of a drop of Laudanum, though Dr. Merce put 15 drops of Laudanum as equivalent to a grain of Opium,) from 3d week to 6 weeks $\frac{1}{100}$ grain, from 6 to 8 weeks $\frac{1}{70}$ grain, and from 2 to 4 months $\frac{1}{40}$ grain." He further says, "the action of a proper dose of Opium is manifested in $\frac{1}{2}$ hour and lasts from 3 to 6 hours; a moderated degree of narcotic action lasting from 6 to 10 hours has never in my experience seemed dangerous *when Opium has been distinctly indicated.*"

Another important rule in the use of Opium in infants is not to repeat the dose at *too short intervals*, and this is a point, we are convinced not sufficiently attended to. It is not to be wondered at, that bad effects have resulted from the large doses of opiates which some practitioners have been in the habit of giving children, and from their frequent repetition. One writer says, in speaking of the dose necessary to be given, "this is not to be repeated in less than one hour." This is too frequent and we should wait longer to see the effects before repeating, for though the first dose may not narcotize, when too frequently repeated, narcotism may occur. If frequently repeated doses do not narcotize, they will injure the tone of the stomach, and the disposition on the part of the child to take nourishment, a matter of great importance in long continued wasting diarrhœa and other depressing diseases.

The form of Opium to be used in young subjects is a matter of importance. Opium, Tinct. Opium, and Paregoric are the best, for they are of known strength, and can be accurately divided. "Syrup of Poppies," much used in England, is not much employed in this country, and should not, on account of its uncertain strength, be advised. We should be cautious in using Laudanum which has been standing long, and having evaporated, leaves an undue quantity of Opium at the bottom of the vial, which, of course may produce fatal effects upon a very young child. The objection to "Dover's Powder" is that the heavier portion of the powder, which is the Sulphate of Potassa, sinks to the bottom, leaving the Opium and Ipecac at the upper portion; this is where it has been standing long, and was first noticed by Dr. Neligan of Dublin. This difficulty may be obviated by shaking it before using, or directing the apothecary to do so.

Every practitioner has been struck with cases evincing the improper use of Opium in children by the non-medical public, all of us have probably seen children who under their habitual use, by over-anxious or careless mothers, become puny and pale, with dry skin, sunken features, with an appearance of premature old age depicted in the face, and with a blank, stupid countenance. It is our duty to apprize mothers of these effects, and warn them against this unjustifiable use of Opium. In a report to the "House of Commons" from the coroners of England and Wales of 543 deaths by poisoning, 52 were from Opium in some form, generally given by mothers or nurses in ignorance of its effects.

A treatise describing with precision the peculiarities of medicinal agents in their action upon infants and their doses has long been needed; there is no such treatise, and the works on "Materia Medica" contain but little information on the subject, and consequently the young practitioner must rely on incidental observations derived from his general works on "Practice of Medicine," and on "Diseases of Children," and the slow accumulations and the painful lessons of his own experience.

